# Interobserver Agreement in Ultrasound Risk Stratification Systems for Thyroid Nodules on Static Images Versus Cine-Loop Video Sequences

**DOI:** 10.3390/diagnostics14192138

**Published:** 2024-09-26

**Authors:** Simone Agnes Schenke, Manuela Petersen, Rainer Görges, Verena Ruhlmann, Michael Zimny, Johannes-Paul Richter, Daniel Groener, Justus Baumgarten, Michael C. Kreissl, Alexander R. Stahl, Michael Grunert, Burkhard Klemenz, Franziska Veit, Georg Zettinig, Philipp Seifert

**Affiliations:** 1 Division of Nuclear Medicine, Department of Radiology and Nuclear Medicine, University Hospital Magdeburg, 39120 Magdeburg, Germany; simoneschenke@web.de (S.A.S.);; 2 Department and Institute of Nuclear Medicine, Hospital Bayreuth, 95445 Bayreuth, Germany; 3 Department of General, Visceral, Vascular and Transplant Surgery, University Hospital Magdeburg, 39120 Magdeburg, Germany; 4 Clinic of Nuclear Medicine, University Hospital Essen, 45147 Essen, Germany; 5 Practice for Nuclear Medicine, 47051 Duisburg, Germany; 6 Practice for Nuclear Medicine, 45136 Essen, Germany; 7 Institute of Nuclear Medicine, 63450 Hanau, Germany; 8 Practice for Nuclear Medicine, AnthroNUK, 13349 Berlin, Germany; 9 Department of Nuclear Medicine, University Hospital Frankfurt, 60590 Frankfurt, Germany; 10 Research Campus STIMULATE, Otto-von-Guericke University, Magdeburg, 39106 Magdeburg, Germany; 11 Institute of Radiology and Nuclear Medicine, RIZ, 86150 Augsburg, Germany; 12 Department of Nuclear Medicine, German Armed Forces Hospital Ulm, 89081 Ulm, Germany; 13 Department of Nuclear Medicine, University Hospital Ulm, 89081 Ulm, Germany; 14 Institute of Radiology and Nuclear Medicine, Dr. von Essen, 56068 Koblenz, Germany; 15 Vienna Thyroid Center Schilddrüsenpraxis Josefstadt, 1080 Wien, Austria; 16 Clinic of Nuclear Medicine, University Hospital Jena, 07747 Jena, Germany

**Keywords:** thyroid nodule, ultrasound, Thyroid Imaging Reporting and Data Systems (TIRADS), interobserver agreement, cine-loop

## Abstract

Purpose: To evaluate the impact of video sequences (cine-loops) on the interobserver agreement (IOA) using risk stratification systems (RSSs) for thyroid nodules (TNs). Methods: Twenty TNs were randomly selected from a large database and evaluated by twelve experienced observers using five different RSSs (Kwak-, ACR-, EU-, Korean-TIRADS, ATA Guidelines). In the first step, the evaluation was conducted based on static ultrasound (US) images in two planes (“static”). Six months later, these cases were reevaluated by the same observers using video sequences in two planes (“cine-loops”). Fleiss’ kappa (κ) was calculated for the IOA analyses. Results: IOA on static was moderate with κ values of 0.46, 0.42, 0.40, 0.45, and 0.38 for the Kwak-, ACR-, EU-, Korean-TIRADS, and ATA Guidelines, respectively, while the IOA on cine-loops was fair with κ values of 0.41, 0.38, 0.37, 0.36, and 0.34 for the Kwak-, ACR-, EU-, Korean-TIRADS, and ATA Guidelines, respectively. The overall IOA was superior in static images versus cine-loops (*p* = 0.024). Among other findings, the subgroup analyses (related to age, gender, US certificates, number of thyroid US per week, and RSSs experience) particularly showed that the experience of the observers in using RSSs had a significant influence on the IOA. Conclusions: The overall IOA (all twelve observers and all five RSSs) was superior on static US images in comparison to cine-loops. Furthermore, the overall IOA of the five US features revealed superior κ values of the static images over cine-loops. However, this impact was significantly lower when the observers were highly experienced in the use of US RSSs of TNs.

## 1. Introduction

Gray scale ultrasound (US) is a standard tool for the assessment of the thyroid gland and is important for the primary diagnosis and follow-up of thyroid diseases.

The advantages of US in comparison to other imaging methods are its excellent resolution and contrast in soft tissue, real-time applicability, rapid feasibility, portability and flexibility, the simple repeatability, the high acceptance by users and patients, and the low procedural costs [1]. Although US has been used to assess thyroid nodules (TNs) for many years, the accuracy in differentiating between benign and malignant lesions based on individual criteria is low [2]. International societies have developed different risk stratification systems (RSSs), known as Thyroid Imaging Reporting and Data Systems (TIRADS), which are based on specific US characteristics (solidity, hypoechogenicity, irregular/microlobulated margin, microcalcification, and taller-than-wide) [3,4,5,6,7,8].

Known disadvantages of conventional US are the intra- and interobserver variability. Studies have shown that deviations in the assessment of the individual sonographic criteria can be considerable [9,10]. Significant interobserver variability has been described for both the volumetric determinations and risk stratification of TNs [10,11]. Therefore, thyroid US is dependent on the specific expertise of the examiners [12,13]. Different users with different levels of experience and missing or inadequate digital documentation restrict the potential of 2D static US, especially in the follow-up. It has already been shown that the interobserver agreement (IOA) increases with the increasing experience of the examiner [13,14]. In most cases, only static image captures are available, and it is impossible to determine in retrospect whether the decisive aspects have been documented.

Modern US devices have a video recording function that enables cine-loops and the transfer of those to the local picture archiving and communication system (PACS). In the PACS, the cine-loops can be scrolled in analogy to CT or MRT series. The same cine-loops of previous examinations of the same patients can be viewed simultaneously and may be used for the assessment of size progression and sonomorphologic change. It is possible to check non-optimally selected parameters for determining the volume and size dynamics of TNs and to repeat risk stratification. Even if relevant lesions are missed during the live US, they can be identified during the follow-up examination. The initial conceptual considerations on this topic have already outlined in detail in the 1990s [15]. Nevertheless, this approach has thus far been implemented in only a limited number of specialties such as cardiology [16]. In numerous medical specialties, cine-loops are either not acquired at all or are acquired and documented without protocols. The use of cine-loops in the context of abdominal ultrasound examinations has been the subject of individual studies [17,18]. The additional value of cine-loops in terms of interobserver variability has already been confirmed in gynecological disorders [19,20]. For the thyroid, there are scarce publications on this topic.

The aim of this study was to evaluate the impact of cine-loops on the IOA using five RSSs for TNs. Particular attention was paid to the comparison on static US images and US cine-loops as well as the experience of the observers.

## 2. Material and Methods

### 2.1. Patients

This pilot study included 20 consecutive adult patients with cytologically or histopathologically diagnosed TNs who presented at the Clinic of Nuclear Medicine in Bayreuth Hospital between November 2022 and February 2023. Bayreuth is a city in Bavaria (Germany) with approximately 75,000 inhabitants. Patients are sent to the Clinic of Nuclear Medicine for thyroid examinations preselected by other outpatient specialists, most frequently by general practitioners.

### 2.2. Inclusion Criteria

- Age ≥ 18 years;
- TNs ≥ 1 cm on thyroid US;
- Fine-needle aspiration cytology (FNAC) and/or histopathological results after thyroid surgery clearly assignable to the included TN.

### 2.3. Exclusion Criteria

- Age < 18 years;
- Patients with a history of thyroid surgery and/or radioiodine therapy;
- Thyroid dysfunction;
- Autoimmune thyreopathy (Graves’ disease, Hashimoto’s thyroiditis);
- Autonomously functioning TNs;
- TNs < 1 cm.

### 2.4. Thyroid Ultrasound Techniques

The indication for or against invasive interventions (FNAC and/or surgery) was determined by clinical standardized diagnostic algorithms according to the current national and international guidelines [5,6,21].

**Figure 1:**
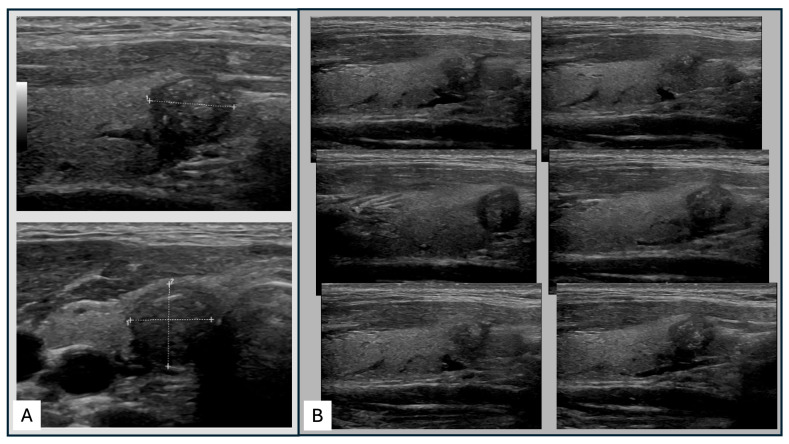
(**A**) Static US images (sagittal and transverse), (**B**) captures from the corresponding US cine-loops (exemplified by a sagittal scan).

The sonographic examinations were performed with a GE Logiq S7 Pro US device equipped with a L3-12 MHz transducer (GE Healthcare, Milwaukee, WI, USA) by one thyroid expert examiner (approx. 20 y of clinical experience in thyroid diagnostics and >10 y of experience with RSS for TNs). The examiner subjectively decided which part of the TNs were captured for the static US images in both the transverse and sagittal orientations. In accordance with the common clinical standard, only one static image was recorded per plane (i.e., a total of two static US images per TN). Special care was taken to depict the representative nodule characteristics and maximum lesion extent. The US cine-loops were not acquired according to a standardized operating procedure (SOP). Standard parameters for the thyroid US presetting (i.e., gain 60, usual frame rate 24–30, THI, crossbeam, double focus) were optimized for each individual patient depending on the location of the nodules of interest. Transverse and sagittal US cine-loops capturing the entire extent of the nodules in both planes were recorded and prepared as replayable videos for the observers (cine-loop time: mean 11.8 ± 4.0 s, range 6.7–22.3 s). An example of an US dataset is shown in Figure 1.

### 2.5. Observers and Ultrasound Assessments

Twelve observers, all members of the German TIRADS Study Group (GTSG; www.tirads.de), with different levels of experience in thyroid US, initially had 4 weeks to assess the US data files of the 20 TNs. Firstly, before any US feature or RSS documentation was conducted, all observers were asked for a purely subjective 5-tier classification (benign, non-suspicious, moderate suspicious, suspicious, highly suspicious) as well as a purely subjective recommendation for or against FNAC, only on the basis of their impressions and clinical experiences. Secondly, all observers classified the TNs according to the Kwak-TIRADS, ACR TI-RADS, EU-TIRADS, updated K-TIRADS, and ATA classifications [4,5,7,8,22]. The following US features were documented by all observers:

Composition (solid, <10, 10–50, 50–90, >90% cystic, spongiform), echogenicity (marked hypoechoic, hypoechoic, isoechoic, hyperechoic, completely cystic), margin (smooth, macrolobulated, microlobulated, irregular, ill-defined, extrathyroidal extension), shape (round, taller-than-wide, wider-than-tall), calcifications/spots (none, colloidal-cystic associated spots, macrocalcifications, rim calcifications, rim calcifications with small extrusive soft tissue component, microcalcifications).

After an interval of 6 months, cine-loops of the same 20 TNs were reassessed by all observers in the same way (first subjective, non-standardized evaluation followed by TIRADS classification). The observers were blinded to all clinical patient data except for the size of the nodules.

All patient data were collected as part of routine clinical practice in accordance with the Declaration of Helsinki and its annexes and were analyzed retrospectively. Therefore, a separate informed consent was waived.

### 2.6. Calculations and Statistical Analyses

Microsoft Excel software version 16.79.1 (Microsoft Corporation, Remond, WA, USA) was used for data storage, all calculations including Fleiss’ kappa (κ) and 95% confidence intervals [95%-CI] as well as creation of the figures. According to Landis and Koch, the respective assessments of the calculated κ values are shown in Table 1 [23].

To analyze the impact of TN sizes and observer characteristics on the IOA, subgroup calculations were conducted. These included: TNs ≤ 2 cm versus TNs > 2 cm, < or ≥than 60 US examinations per week, ≤ or > than 3 years of RSS experience. The 95%-CI were considered for comparisons between κ values. A significant difference was assumed when the lower 95%-CI of one value did not overlap the upper 95%-CI of the comparative value. The Mann–Whitney U test was used for comparisons of metric parameters other than Fleiss’ kappa, *p*-values <.05 were considered significant.

Cutoff values between benign and malignant for performance calculations were defined at suspicious, 4c, TR5, 5, high, and high for the subjective scale, Kwak-TIRADS, ACR TI-RADS, EU-TIRADS, Korean-TIRADS, and ATA Guidelines, respectively.

## 3. Results

The N = 20 rated TNs included N = 6 papillary thyroid carcinomas (N = 5 classical, N = 1 follicular variant), N = 13 benign thyroid adenomas, and N = 1 benign intrathyroidal parathyroid adenoma. The mean nodule size was 25 ± 10 mm (median: 23 mm; range: 10–55 mm). The mean age of patients were 50 ± 11 years (male) and 43 ± 6 years (female).

The twelve observers (N = 3 female) were recruited from nine different German institutions and one Austrian outpatient clinic. All observers had at least three years of experience with US RSS for TNs and were familiar with all included systems. However, in daily routine, the most clinical experience is available for the following systems (listed in descending order): Kwak-TIRADS, ACR TI-RADS, and ATA Guidelines. The observer characteristics are listed in Table 2.

### 3.1. All Observers

In general, the overall IOA (all twelve observers and all five RSSs) was superior on static US images in comparison to cine-loops (*p* = 0.024). Fair to moderate agreement was obtained with the best results for Kwak-TIRADS on static US images (κ = 0.46) and the worst results for the ATA Guidelines on US cine-loops (κ = 0.34). For Korean-TIRADS, superior IOA was found on static US images in comparison to the US cine-loops. Detailed results are shown in Table 3.

No statistically significant differences in the overall IOA between static US images and US cine-loops were obtained regarding the RSS-based recommendations for or against FNAC. However, ATA revealed distinctly inferior IOA data in comparison to the other investigated RSSs. Detailed results are shown in Table 4.

Furthermore, for all TNs, the observers were asked to provide a purely subjective scale without considering qualified US features or RSSs. The κ values revealed slight agreement for both static US images (0.20 [0.18–0.23]) and US cine-loops (0.18 [0.16–0.21]). These were therefore clearly inferior to the IOA of RSSs. However, the purely subjective recommendations for or against FNAC was different. The κ values revealed fair agreement for both static US images (0.40 [0.35–0.46]) and US cine-loops (0.39 [0.33–0.44]) and were comparable to the IOA regarding the recommendations of the RSS.

The overall IOA of the US features revealed superior κ values of the static US images over US cine-loops (*p* = 0.047). The IOA of most single US features were superior on static US versus US cine-loops. Particularly high differences were found for the assessment of the TN form. Detailed results are shown in Table 5.

The overall IOA was superior for TNs ≤ 2 cm versus TNs > 2 cm. Static US images were superior to US cine-loops in TNs ≤ 2 cm for ACR TI-RADS and Korean-TIRADS. Detailed results are shown in Table 6 and Table 7.

### 3.2. Subgroup “US Per Week”

When considering the number of thyroid US the observers performed per week in 2023, the following IOA comparisons revealed significant differences (detailed results are shown in Table 8):

- <60 (static): 0.46 ± 0.03 *versus* < 60 (loops): 0.39 ± 0.04, *p* = 0.024;
- ≥60 (static): 0.44 ± 0.05 *versus* ≥ 60 (loops): 0.37 ± 0.02, *p* = 0.014.

### 3.3. Subgroup “RSS Experience”

**Figure 2:**
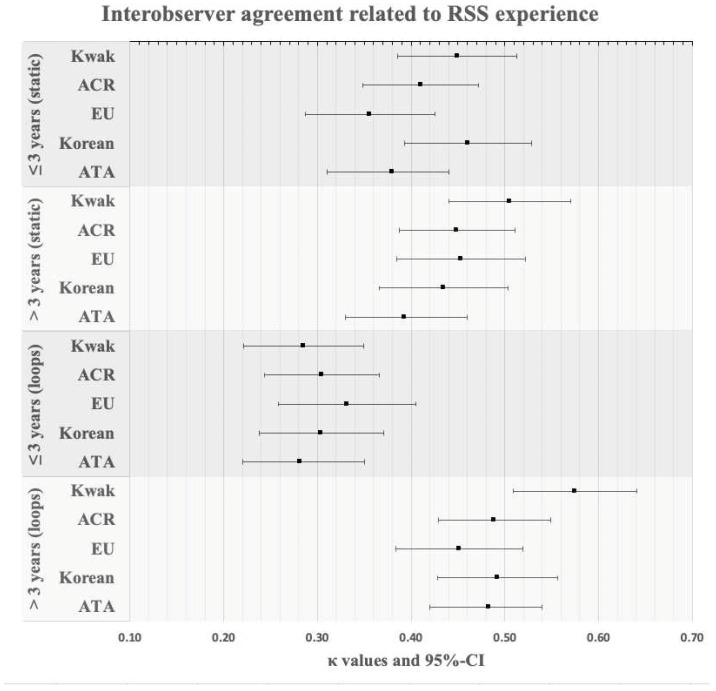
Interobserver agreement related to RSS experience. Abbreviations: RSS—risk stratification system, ACR—American College of Radiology, EU—European Union, ATA—American Thyroid Association.

When considering an observer-related RSS experience cutoff of 3 years, the following IOA comparison revealed significant differences (detailed results are shown in Table 9 and Figure 2):

- ≤3 years (static): 0.41 ± 0.04 *versus* ≤ 3 years (loops): 0.30 ± 0.02, *p* = 0.006;
- ≤3 years (loops): 0.30 ± 0.02 *versus* > 3 years (loops): 0.50 ± 0.05, *p* = 0.006;
- ≤3 years (static Kwak): 0.45 [0.39–0.51] *versus* ≤ 3 years (loops Kwak): 0.29 [0.22–0.35];
- ≤3 years (static Korean): 0.46 [0.39–0.53] *versus* ≤ 3 years (loops Korean): 0.30 [0.24–0.37];
- ≤3 years (loops Kwak): 0.29 [0.22–0.35] *versus* > 3 years (loops Kwak): 0.58 [0.51–0.64];
- ≤3 years (loops ACR): 0.30 [0.24–0.37] *versus* > 3 years (loops ACR): 0.49 [0.43–0.55];
- ≤3 years (loops Korean): 0.30 [0.24–0.37] *versus* > 3 years (loops Korean): 0.49 [0.43–0.56];
- ≤3 years (loops ATA): 0.28 [0.22–0.35] *versus* > 3 years (loops ATA): 0.48 [0.42–0.54].

### 3.4. Diagnostic Performance

The overall IOA was superior for the N = 14 benign TNs (static US images: 0.42 ± 0.6, US cine-loops: 0.35 ± 0.5) in comparison to the N = 6 malignant TNs (static US images: 0.22 ± 1.0, US cine-loops: 0.30 ± 0.2). However, due to the low number of malignant TNs, this comparison should neither be considered for reliable statistical analyses nor for respective conclusions. The comprehensive performance results of all observers are shown in Table 10. The inferior values for the ATA Guidelines mainly resulted from the relatively high number of “not applicable” ratings. In 41 (static US images) and 43 (US cine-loops) out of 240 respective ratings (20 TNs rated by 12 observers), the ATA was not applicable due to the known limitations [10].

## 4. Discussion

The results of US investigations of TNs are still often documented by static image captures, with the operator determining which features are worthy of documentation. However, a limitation of conventional US lies in its reliance on the observer. US cine-loops can be used to retrospectively determine whether the pathological features have been recorded properly or if relevant findings are missing. The IOA and the impact of consensus reading using four different RSSs for TNs based on static captures have already been analyzed by our group [10].

The current study analyzed the impact of cine-loops on the IOA of five RSSs for TNs on static images versus cine-loop video sequences. To the best of our knowledge, there are scarce publications on this topic.

The results showed that, in the purely subjective assessment without consideration of RSSs, only slight agreement between the observers was found for both static US images and US cine-loops. The utilization of RSSs served to diminish the influence of subjectivity. Notably, the results for purely subjective recommendations for or against FNAC were different. The κ values revealed fair agreement for both static US images and US cine-loops and were comparable to the IOA regarding the recommendations of the RSS. The consistency of the results may possibly indicate the need for additional improvements or the standardization of the RSS criteria in order to increase the IOA. The data showed a notably lower IOA noted with the ATA Guidelines in comparison to other RSSs when recommending FNAC. A weakness in the ATA Guidelines is the fact that isoechoic solid nodules with further suspicious US features are not assigned to any of the classifications. The agreement of all observers for RSSs was obtained with the best results for Kwak-TIRADS on both static US images and cine-loops, probably because it is a simple system with few options and had been used by most observers in our group for the longest time. In general, the overall IOA (all twelve observers and all five RSSs) was significantly superior on static US images in comparison to cine-loops. The overall IOA of the US features revealed superior κ-values of the static US images over the US cine-loops, and the IOA of most single US features were superior on static US versus US cine-loops. Particularly high differences were found for the assessment of the form of the TNs. Given that the examiner performing the US examinations was highly experienced, it can be assumed that the decisive parameters were accurately recorded on the static image. In contrast, in the cine-loop data, the observers had to search for the decisive parameters themselves. The performance of the observers and any potential biases may have been impacted by their differing degrees of clinical familiarity with various RSSs.

Słowińska-Klencka et al. investigated the impact of real-time (rt) US vs. static US on the categorization of TNs in EU-TIRADS. Three experienced raters assessed 842 TNs on rtUS and reassessed them by the use of static US images. Reproducibility of the sonographic features and classification of TNs was estimated with Krippendorff’s alpha coefficient (Kα). The reproducibility of EU-TIRADS categories on static US in relation to rtUS was 70.9–76.5% for all raters (Kα: 0.60–0.68), with the lowest reproducibility for category 5 (48.7–77.8%) and highest for category 3 (80.0–86.5%). Microcalcifications were not identified in the static images, and the reproducibility varied for marked hypoechogenicity; 12.5–84.6%, Kα: 0.14–0.48. [24].

In comparison, Bae et al. evaluated the IOA for 253 TNs between rtUS assessment and retrospective US interpretation by using K-TIRADS [25]. Each US examination was performed by a single radiologist with more than 8 years of experience in thyroid imaging. Then, the same radiologist prospectively evaluated the US images of the TNs. The static US images were retrospectively evaluated by another radiologist with 2 years of experience, who had not been involved in the rtUS examinations. They found the overall IOA to be almost perfect for orientation (κ: 0.868), substantial for spongiform appearance (κ: 0.786), calcification (κ: 0.778), composition (κ: 0.754), echogenicity (κ: 0.747), shape (κ: 0.670), margin (κ: 0.666), and final K-TIRADS categories (κ: 0.754), respectively. The IOA for predominantly cystic composition and ill-defined margin were relatively low in this study. They explained that the volumetric amount of the cystic composition was difficult to accurately evaluate on the static images and that spiculated margins could also be misinterpreted as ill-defined margins on the static images. Overall, it seems that the more descriptive a sonography feature, the lower the IOA. In comparison, our results showed, for both static US images and US cine-loops, the lowest overall IOA for margins and calcifications and the highest overall IOA for shape.

Solymosi et al. chose a different approach [26]. After the blinded online evaluation of video recordings of the US examinations of 123 nodules, seven experts from seven centers answered 17 TIRADS-related questions. Examination of the video recordings revealed substantially different IOA in the interpretation of four US features (presence of microcalcifications, irregular margins, extrathyroidal extension, iso-, hyper-, or hypoechogenic appearance, and if hypoechogenic whether minimally, moderately, or very hypoechogenic). Interobserver variations were compared using Gwet’s AC1 interrater coefficients; higher values mean better concordance (maximum 1.0). The values were 0.34 for irregular margins, 0.53 for microcalcifications, 0.72 for echogenicity, and 0.79 for extrathyroidal extension, respectively. They showed that the smaller the nodule size, the better the IOA is for the determination of echogenicity. On the other hand, they also observed that the larger the nodule size, the better the IOA becomes in terms of the detection of microcalcifications. In comparison, the overall IOA in our results was significantly superior for TN ≤ 2 cm versus TN > 2 cm, and static US images were significantly superior to US cine-loops in TN ≤ 2 cm for ACR TI-RADS and Korean-TIRADS.

In our study, when considering the number of thyroid US the observers performed per week (for both <60 and >60 US per week), the IOA was significantly superior for static US images versus US cine-loops. When considering an observer-related RSS experience cutoff of 3 years, the IOA comparisons revealed significant differences. The data suggest that the more experienced the observer, the more effectively the US cine-loops can be used. Otherwise, another reason could be the fact that the initial training for evaluating TNs according to RSSs is conducted with static images.

Similar results in other organ systems were shown in another study. Parsai et al. conducted a comparative study to assess the diagnostic value of sonographic examinations acquired with a standardized video clip approach in comparison to examinations performed with static images alone in 60 patients with various hepatic and extrahepatic pathologies [27]. The research group described that the use of video clips improved diagnostic accuracy compared with static images alone, and that video sequences were not reliant on the operator and offered greater objectivity compared to still images. Additionally, the interpretation of videos clips did not result in significant clinical errors. When using static images alone, all observers (regardless of their US experience) missed focal lesions in many cases.

To our knowledge, no consensus has been reached to standardize US cine-loop video sequences of the thyroid thus far. Seifert et al. first introduced an US cine-loop standard operating procedure (SOP) and mentioned that the image quality of cine loops was usually lower than that of static images. Possible reasons could include cropped cranial and caudal poles, movement of the thyroid gland, artifacts caused by inadequate application of US gel, and rapid movement of the US probe [28].

In the future, interobserver variability could be minimized by software systems that support the physician, and the acquired data could be standardized by the use of three dimensional tomographic ultrasound [29]. Advancements in medical technology, such as improved US devices, structured reports, and the integration of artificial intelligence, could enhance the precision of the cine-loops. Artificial intelligence, in particular, has already demonstrated its value in evaluating TNs [30,31].

### Limitations

The number of patients included was relatively low and the reliability of the presented data needs to be proven by future research containing larger patient collectives. However, the objective of the authors was to obtain preliminary insights into the impact of US cine-loops on interobserver variability in the assessment of TNs within a concise timeframe and with a substantial number of experienced observers.

It should be noted that this study was subject to a selection bias with a relatively high malignancy rate of 30%. Furthermore, a parathyroid adenoma was included for which the included RSSs were not evaluated. However, parathyroid adenomas appearing within the thyroid parenchyma are part of the clinical reality. The aim of this study was not to evaluate the performance of the RSSs, but the interobserver variability, so we decided to include this node according to the predefined inclusion and exclusion criteria. In future study protocols, parathyroid adenomas will be an exclusion criterion from the outset.

The application and documentation of static US images and US cine-loops were not performed according to a SOP. However, there are no established recommendations in the international guidelines thus far, and the pilot character of the presented study reflects the need for more structured data in this manner. The consistency of the quality of the included image and video data was ensured by the fact that only one very experienced examiner recorded the US examinations with a well-known US device.

## 5. Conclusions

The overall interobserver agreement was superior on the static ultrasound images in comparison to the ultrasound cine-loops for the assessment of thyroid nodules. However, this impact was significantly lower when the observers were highly experienced in the use of ultrasound risk stratification systems. Standardized operating procedures for the acquisition of ultrasound cine-loops should be investigated in larger patient cohorts.

## Figures and Tables

**Table 1:** Assessment of κ values.

κ	Assessments
0	No agreement
0–0.20	Slight agreement
0.21–0.40	Fair agreement
0.41–0.60	Moderate agreement
0.61–0.80	Substantial agreement
0.81–1	Almost perfect agreement
1	Complete agreement

**Table 2:** Observer Characteristics.

#	Age (y)	Sex	Clinical xp. (y)	Institution	Specialization	US Certificates	Thyroid US Per Week	RSS xp. (y)
1	55	m	25	Practice	Nuclear medicine and radiology	None	N~70	3
2	61	m	31	Hospital	Nuclear medicine	DEGUM ENT, courses in internal medicine, and surgery	N~30	6
3	39	m	10	University hospital	Nuclear medicine	None	N~55	5
4	40	f	13	Practice	Nuclear medicine	Course in internal medicine	N~80	3
5	54	m	29	Practice	Nuclear medicine	ÖGUM III	N~60	3
6	43	m	15	Practice	Nuclear medicine and radiology	DEGUM abdominal, DEGUM musculoskeletal	N~100	3
7	32	m	7	University hospital	Nuclear medicine	None	N~45	5
8	51	f	18	University hospital	Surgery	None	N~10	3
9	43	m	16	Hospital	Nuclear medicine	None	N~30	6
10	57	m	30	Practice and hospital	Nuclear medicine	None	N~150	12
11	63	m	32	Practice and university hospital	Nuclear medicine	DEGUM ENT, course in abdominal US	N~60	11
12	42	f	16	Practice and university hospital	Nuclear medicine	None	N~55	3

Abbreviations: y—years, xp.—experience, US—ultrasound, RSS—risk stratification system, m—male, N~—approximate number, DEGUM—Deutsche Gesellschaft für Ultraschall in der Medizin, ENT—Ear, Nose, and Throat/otolaryngology, f—female, ÖGUM—Österreichische Gesellschaft für Ultraschall in der Medizin.

**Table 3:** Interobserver agreement of all observers.

RSS	Static US Images κ [95%-CI]	US Cine-Loops κ [95%-CI]	Comparison
Kwak	0.46 [0.43–0.49]	0.41 [0.38–0.44]	No statistical difference
ACR	0.42 [0.39–0.45]	0.38 [0.35–0.41]	No statistical difference
EU	0.40 [0.37–0.43]	0.37 [0.34–0.40]	No statistical difference
Korean	0.45 [0.42–0.48]	0.36 [0.33–0.40]	Static > loops
ATA	0.38 [0.35–0.41]	0.34 [0.31–0.37]	No statistical difference
mean ± SD	0.39 ± 0.03	0.37 ± 0.03	Static > loops, *p* = 0.024

Abbreviations: RSS—risk stratification system, ACR—American College of Radiology, EU—European Union, ATA—American Thyroid Association, SD—standard deviation, US—ultrasound, κ—Fleiss’ kappa, CI—confidence interval.

**Table 4:** Interobserver agreement of all observers for the several risk stratification systems’ recommendations regarding fine-needle aspiration cytology (excluding Kwak-TIRADS).

RSS FNAC	Static US Images κ [95%-CI]	US Cine-Loops κ [95%-CI]	Comparison
ACR	0.44 [0.39–0.49]	0.50 [0.45–0.56]	No statistical difference
EU	0.43 [0.38–0.48]	0.47 [0.42–0.52]	No statistical difference
Korean	0.45 [0.42–0.48]	00.44 [0.39–0.50]	No statistical difference
ATA	0.19 [0.13–0.24]	0.12 [0.06–0.17]	No statistical difference
mean ± SD	0.39 ± 0.13	0.38 ± 0.18	No statistical difference

Abbreviations: RSS—risk stratification system, FNAC—fine-needle aspiration cytology, ACR—American College of Radiology, EU—European Union, ATA—American Thyroid Association, SD—standard deviation, US—ultrasound, κ—Fleiss’ kappa, CI—confidence interval.

**Table 5:** Interobserver agreement of all observers for the ultrasound features.

US features	Static US Images κ [95%-CI]	US Cine-Loops κ [95%-CI]	Comparison
Composition	0.43 [0.40–0.46]	0.36 [0.33–0.39]	Static > loops
Echogenicity	0.44 [0.40–0.47]	0.29 [0.25–0.33]	Static > loops
Borders	0.30 [0.27–0.33]	0.28 [0.25–0.31]	No statistical difference
Calcifications	0.31 [0.28–0.34]	0.25 [0.22–0.28]	Static > loops
Form	0.76 [0.71–0.81]	0.40 [0.36–0.44]	Static >> loops
Mean ± SD	0.45 ± 0.19	0.32 ± 0.06	Static > loops, *p* = 0.047

Abbreviations: US—ultrasound, SD—standard deviation, κ—Fleiss’ kappa, CI—confidence interval.

**Table 6:** Interobserver agreement of all observers for risk stratification systems of thyroid nodules ≤ 2 cm (N = 9).

RSS of TNs ≤ 2 cm	Static US images κ [95%-CI]	US Cine-Loops κ [95%-CI]	Comparison
Kwak	0.50 [0.45–0.55]	0.41 [0.36–0.46]	No statistical difference
ACR	0.55 [0.49–0.61]	0.38 [0.33–0.43]	Static > loops
EU	0.48 [0.42–0.53]	0.38 [0.33–0.43]	No statistical difference
Korean	0.55 [0.50–0.61]	0.36 [0.31–0.41]	Static > loops
ATA	0.45 [0.39–0.50]	0.35 [0.30–0.40]	No statistical difference
mean ± SD	0.51 ± 0.04	0.38 ± 0.02	Static > loops, *p* = 0.006

Abbreviations: RSS—risk stratification system, TNs—thyroid nodules, ACR—American College of Radiology, EU—European Union, ATA—American Thyroid Association, SD—standard deviation, US—ultrasound, κ—Fleiss’ kappa, CI—confidence interval.

**Table 7:** Interobserver agreement of all observers for risk stratification systems of thyroid nodules > 2 cm (N = 11).

RSS of TNs > 2 cm	Static US Images κ [95%-CI]	US Cine-Loops κ [95%-CI]	Comparison
Kwak	0.34 [0.29–0.38]	0.35 [0.29–0.42]	No statistical difference
ACR	0.31 [0.27–0.35]	0.34 [0.29–0.40]	No statistical difference
EU	0.27 [0.23–0.32]	0.27 [0.21–0.33]	No statistical difference
Korean	0.22 [0.18–0.26]	0.18 [0.13–0.24]	No statistical difference
ATA	0.22 [0.18–0.26]	0.23 [0.17–0.28]	No statistical difference
mean ± SD	0.27 ± 0.05	0.27 ± 0.08	No statistical difference, *p* = 0.417

Abbreviations: RSS—risk stratification system, TNs—thyroid nodules, ACR—American College of Radiology, EU—European Union, ATA—American Thyroid Association, SD—standard deviation, US—ultrasound, κ—Fleiss’ kappa, CI—confidence interval.

**Table 8:** Averaged interobserver agreement related to number of US per week performed by the observers.

Subgroups		κ Mean ± SD	*p*-Values	
I	<60 (static), N = 6	0.46 ± 0.03	I vs. II	0.233
II	≥60 (static), N = 6	0.44 ± 0.05	I vs. III	0.024
III	<60 (loops), N = 6	0.39 ± 0.04	III vs. IV	0.417
IV	≥60 (loops), N = 6	0.37 ± 0.02	II vs. IV	0.014

Abbreviations: κ—Fleiss’ kappa, SD—standard deviation, N—number, vs.—versus.

**Table 9:** Averaged interobserver agreement related to the observers’ RSS experience.

Subgroups		κ Mean ± SD	*p*-Values	
I	≤3 years (static), N = 6	0.41 ± 0.04	I vs. II	0.200
II	>3 years (static), N = 6	0.45 ± 0.04	I vs. III	0.006
III	≤3 years (loops), N = 6	0.30 ± 0.02	III vs. IV	0.006
IV	>3 years (loops), N = 6	0.50 ± 0.05	II vs. IV	0.072

Abbreviations: κ—Fleiss’ kappa, SD—standard deviation, N—number, vs.—versus.

**Table 10:** Diagnostic performance of the examined risk stratification systems as well as the subjective scale.

RSS	Static US Images	US Cine-Loops
Kwak	ACC 76% SEN 58%, SPE 83%, PPV 60%, NPV 82%	ACC 76% SEN 56%, SPE 85%, PPV 62%, NPV 82%
ACR	ACC 75% SEN 63%, SPE 80%, PPV 58%, NPV 83%	ACC 71% SEN 50%, SPE 80%, PPV 52%, NPV 79%
EU	ACC 72% SEN 76%, SPE 70%, PPV 52%, NPV 87%	ACC 70% SEN 61%, SPE 73%, PPV 49%, NPV 81%
Korean	ACC 76% SEN 58%, SPE 84%, PPV 61%, NPV 82%	ACC 78% SEN 40%, SPE 87%, PPV 65%, NPV 82%
ATA	ACC 59% SEN 58%, SPE 60%, PPV 38%, NPV 77%	ACC 61% SEN 51%, SPE 65%, PPV 39%, NPV 76%
Subjective scale	ACC 76% SEN 61%, SPE 83%, PPV 60%, NPV 83%	ACC 77% SEN 51%, SPE 88%, PPV 64%, NPV 81%

Abbreviations: RSS—risk stratification systems, ACR—American College of Radiology, EU—European Union, ATA—American Thyroid Association, ACC—diagnostic accuracy, SEN—sensitivity, SPE—specificity, PPV—positive predictive value, NPV—negative predictive value, US—ultrasound.

## Data Availability

The data that support the findings of this study are available from the corresponding author upon reasonable request.
